# The implementation of low instantaneous dose rate total body irradiation with linear accelerator in small‐size treatment rooms

**DOI:** 10.1002/acm2.14502

**Published:** 2024-09-04

**Authors:** Zhengxin Gao, Qiuyi Xu, Fengjiao Zhang, Yaling Hong, Qiaoying Hu, Qi Yu, Shen Fu, Qing Gong

**Affiliations:** ^1^ Department of Radiation Oncology Shanghai Concord Medical Cancer center Shanghai China; ^2^ Proton & Heavy Ion Medical Research Center, State Key Laboratory of Radiation Medicine and Protection Soochow University Soochow Jiangsu China

**Keywords:** in‐vivo dosimetry, low instantaneous dose rate total body irradiation, TPS model configuration

## Abstract

**Purpose:**

This paper describes the implementation of an instantaneous low‐dose‐rate total body irradiation (TBI) technique using block‐filtered 6 MV X‐rays with a linear accelerator (LINAC) to reduce pulmonary toxicity.

**Methods:**

In the absence of dedicated TBI‐specific meter‐set dose rates in LINAC and sufficient treatment room size, a 2‐cm‐thick transmission block was used together with a 200‐cm source‐to‐surface distance (SSD) to reduce the instantaneous dose rates of 6 MV x‐rays down to 10 cGy/min, thus alteration to the beam properties. A TBI‐specific dose calculation model was built with data acquired at the treatment planning system (TPS)‐permitted maximum 140‐cm SSD and was validated in phantoms at a 180‐cm SSD. As for planning strategies, we adopted large anterior‐to‐posterior/posterior‐to‐anterior (AP/PA) open fields with multi‐leaf collimator shielding for lungs to achieve target coverage, lung protection, and efficient dose delivery. A custom‐designed sliding couch (Patent No. ZL202123085880.1) was manufactured to support patients during treatment. Measures to control the quality and safety of TBI treatment include machine interlocks, pretreatment checklists, and in‐vivo dose monitoring.

**Results:**

The instantaneous dose rate of block‐filtered 6MV X‐ray was reduced to approximately 7.0 cGy/min at 12.5–7.5 cm depth with a 185–200 cm SSD. The dose calculated by TPS differs from the measurements by 0.15%–1.55% in the homogeneous phantom and 1.2%–4.85% in the CIRS thorax phantom. The open‐field TBI technique achieved V_90%_ (PTV) ≈ 96.8% and MLD = 6.6 Gy with 1‐h planning and 50‐min beam delivery in a single fraction. From February 2021 to July 2023, 30 patients received TBI treatments in our center, and in‐vivo monitoring results differed from TPS calculations by −1.49%–2.10%. After 6–12 months of follow‐ups, all the patients treated in our center showed no pulmonary toxicities of grade 2 or higher.

**Conclusion:**

A low instantaneous dose rate TBI technique can be implemented in the clinic.

## 1 INTRODUCTION

Total body irradiation (TBI) is indeed a critical component in the preparative regimen for bone marrow transplantation.^1^, ^2^, ^3^ Its primary goals are twofold: to eradicate malignant cells that may be resistant to chemotherapy and to immunosuppress the patient sufficiently to allow for the engraftment of donor stem cells. Clinically, TBI can be delivered in two typical regimens: single‐fraction TBI (a total of 3–4 Gy delivered in one fraction) or hyper‐fraction TBI (10–12 Gy delivered in 5 or 6 fractions, BID, 6 h apart, over 2 or 3 consecutive days). Regardless of the treatment regimen, it involves exposing the entire body to mega‐voltage photon beams, and all organs‐at‐risk (OAR) receive a uniform dose. This uniform exposure could lead to significant side effects and complications in radiosensitive ones, particularly in the lungs, causing pulmonary toxicity (PT), which can severely impact respiratory function and, in severe cases, lead to mortality.

In our center, we prepared to deliver hyper‐fraction TBI treatment (8–10 Gy in 4 or 5 fractions, twice daily, 6 h apart, on 2 or 3 consecutive days) using a Linear Accelerator (LINAC). A reduced total dose of 8–10 Gy for TBI is administrated in conjunction with chemotherapy, taking into account the patient's poor performance status, age, and cumulative chemotherapy exposures.^4^ By conducting this treatment, our major concern with this treatment is the incidence of PT following TBI. To mitigate it, it is crucial to reduce both the total dose and instantaneous dose rates to the lungs simultaneously.^5^

Lung shielding is important in TBI treatment. In 1996, Gore's study first revealed that higher radiation doses to the lungs could damage long‐term pulmonary functions.^6^ Additionally, Sampath's study^7^ in 2004 reported that 50% lung‐dose shielding, which means mean lung dose (MLD) ≤6 Gy here, could reduce the incidence of IP from 11% (no shielding) to 2.3% (lung shielding) in hyper‐fractionated TBI (12 Gy in 6 fractions, BID). Recent studies^8^ also reported a higher risk of lung toxicity with MLD > 8 Gy after hyper‐fractionated TBI. Based on the previous study we aim to constraint the MLD ≤7 Gy with multi‐leaf collimators (MLC).

Meanwhile, the ACR‐ASTRO guideline^9^ also suggests using relatively low dose rates to mitigate PT in patients undergoing hyper‐fractionated TBI. Several studies over the last few decades have proved the dose‐rate effect on PT^10^, ^11^, ^12^, ^13^, ^14^ following hyper‐fractionated TBI treatment. Carruthers's study in 2004^10^ found that patients who received hyper‐fractionated TBI (12 Gy in 6 fractions, BID, over 3 days) using a dose rate of 15 cGy/min had a higher rate of PT than those using a dose rate of 7.5 cGy/min. In 2019, Gao's study^11^ reported that patients who received hyper‐fractionated TBI (13.2 Gy in eight fractions, BID, over 4 days) using dose rates ≤15 cGy/min had a lower incidence of PT and improved overall survival as compared to those receiving dose rates ≥15 cGy/mi.

Even though, it remains challenging to determine the optimal dose rate to reduce PT. Gogna's study in 1992 concluded that dose rates ≤8.9 cGy/min would not influence PT for hyper‐fractionated TBI,^15^ while more recent studies^1^, ^5^, ^10^, ^11^ suggested that hyper‐fractionated TBI using dose rates ≤15 cGy/min could decrease the occurrence of PT. Also, Oya's study^16^ reported no significant difference in PT between hyper‐fractionated TBI (12 Gy in 6 fractions, BID, consecutive 3 days) using dose rates of 8 and 19 cGy/min. These findings suggest a safe threshold for dose rates to lungs during hyper‐fractionated TBI treatment ranges from 8 to 19 cGy/min. Although lowering the instantaneous dose to the lungs is safer, it also extends the treatment time, increasing the treatment risks. Therefore, a balance between treatment safety and delivery efficiency is necessary. Considering that TBI treatments have been conducted in a large open field at extended source‐to‐distance (SSD) safely over the past few decades, as reported in AAPM Report No. 17, with low instantaneous dose rates ≤approximately 10 cGy/min, we propose to use instantaneous dose rates ≤10 cGy/min as the threshold for a relatively safe hyper‐fractionated TBI in our study.

In this study, we aim to develop a hyper‐fractionated TBI technique that delivers a total dose of 8–10 Gy in 4–5fractions, twice daily, 6 hours apart, with a low MLD ≤ 7 Gy and a low instantaneous dose rate ≤10 cGy/min to reduce the incidence of PT and improve treatment efficiency simultaneously.

## 2 MATERIALS AND METHODS

### 2.1 Preparation of LINAC's dose rate

#### 2.1.1 Transmission block

Regarding setups, the low instantaneous dose rate (≤10 cGy/min) is typically achieved by using large open fields at an extended SSD of around 400 cm, employing low machine meter‐set dose rates, or sometimes a combination of both. Patients were generally irradiated in anterior‐to‐posterior/posterior‐to‐anterior (AP/PA) lying positions^17^, ^18^, ^19^ or AP/PA standing positions,^20^ with lungs shielding using a customized block.^21^ However, in the absence of dedicated TBI‐specific meter‐set dose rates in our LINAC (Trilogy, Varian Medical Systems, Palo Alto, CA) and limited room size in our center, we employed a transmission block and positioned patients at extended SSDs of 180–200 cm to achieve low instantaneous dose rates ≤10 cGy/min.

**FIGURE 1:**
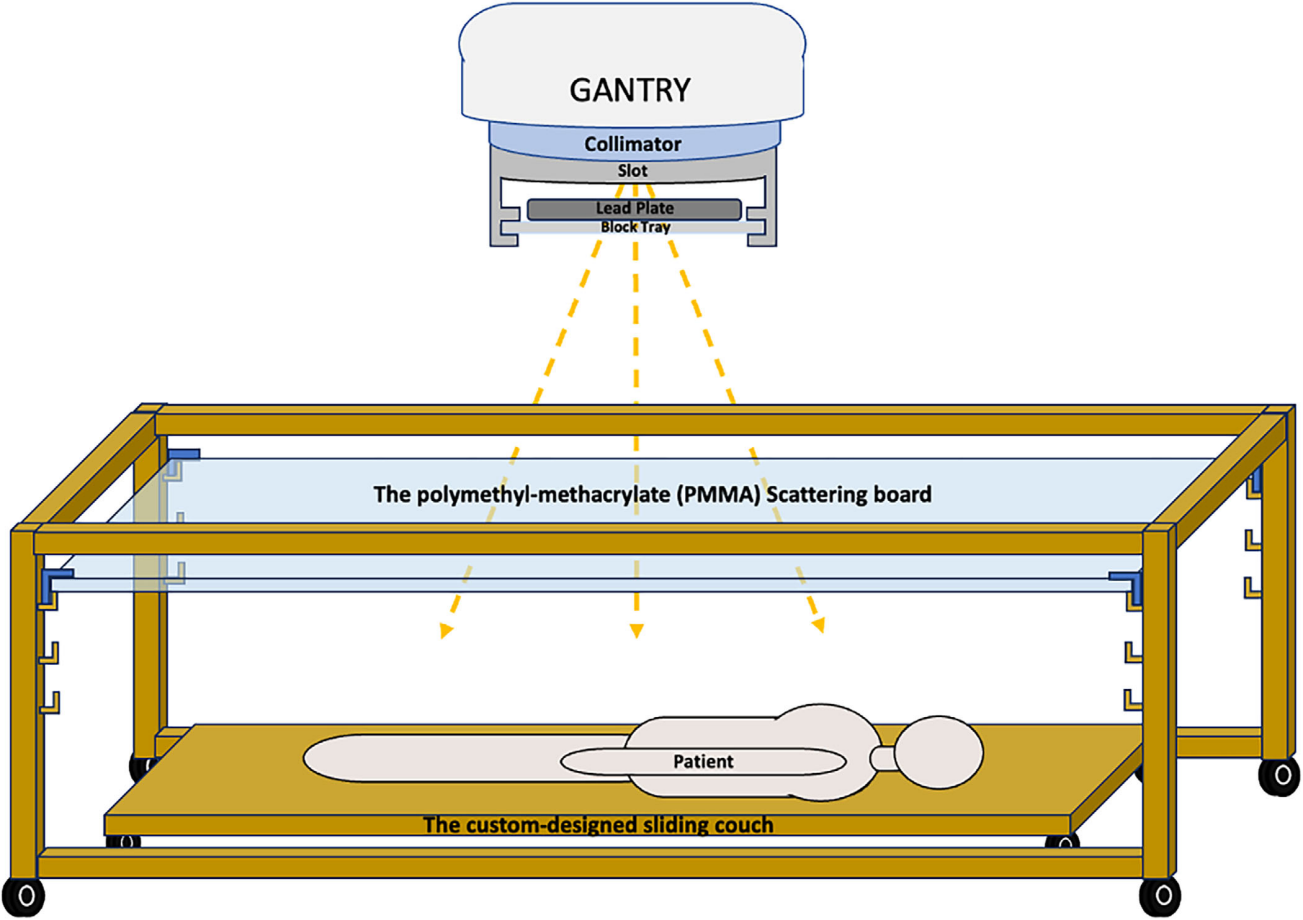
The TBI setup has a transmission block, TBI‐dedicated block tray, slot, and custom‐designed sliding couch system. The transmission block was screwed in the TBI‐dedicated block tray with TBI‐specific custom coding, which was identified by the slot.

As calculated using lead attenuation coefficients, we manufactured a 2‐cm thick, 28 cm × 28 cm wide lead block to achieve lower instantaneous dose rates ≤10 cGy/min in the LINAC. To ensure the safety and proper repositioning of the transmission block, we implemented several measures, including machine interlocks, pre‐treatment checklists, and in‐vivo dose monitoring. For machine interlocks, the transmission block was screwed on the TBI‐dedicated block tray with TBI‐specific customer coding, which is added to each field during TBI planning in the TPS. The block's secure attachment to the tray addresses repositioning and falling concerns. Before each fraction, the TBI‐dedicated block tray is mounted in the slot and identified by the LINAC, as illustrated in Figure 1. Before beam on, therapists must confirm this TBI‐specific customer coding on the treatment console. The LINAC will interlock the beam immediately if the block tray is not correctly positioned or if the wrong block is used. Additionally, an in‐room timeout checklist guides block placement and patient setup before beam‐on, ensuring treatment safety under the supervision of attending therapists, physicists, and oncologists.

The block‐filtered dose rates of 6 MV x‐rays were measured using a calibrated ion chamber (PTW, Freiburg, German) at patients’ midplane (15.0/12.5/10.0/7.5 depth) at treatment SSDs of 185, 190, 195, and 200 cm in a solid water phantom, using LINAC's minimum meter‐set dose rate of 100 MU/min. Similarly, its beam quality index (TPR_20_/TPR_10_) was measured in water with a 10 × 10 cm^2^ field size at 100 cm source‐to‐axis distance (SAD).

#### 2.1.2 A custom‐designed sliding couch system

A custom‐designed sliding couch system (Patent No. ZL202123085880.1), elevated 15 cm over the floor, was manufactured to support patients during the treatment (Figure 1). The sliding couch system is constructed from wood to minimize backscattering as low as possible. Its lateral position is verified with external lasers on the walls, while its longitudinal position is aligned with the optical distance indicator (ODI) projection. The estimated uncertainty of the positioned sliding couch is approximately 1 mm in the lateral direction and 2 mm in the longitudinal direction, owing to the laser width of about 1 mm laterally and the 2 mm width of the ODI projection at 180 cm SSD longitudinally. Once the sliding couch position is determined, therapists lock the wheels at the bottom to prevent any movement during treatment. A 1‐cm‐thick polymethyl‐methacrylate (PMMA) plate was designed to bring the d_max_ of the block‐filtered beam closer to the skin surface and should be placed as close as possible to the patients.

### 2.2 Preparation of TBI‐specific dose calculation model

#### 2.2.1 Model configuration

While the transmission block (as discussed in Section 2.1) invalidated the pre‐configured dose calculation model in TPS (Eclipse, Anisotropic Analytic Algorithm, Varian Medical Systems, Palo Alto, CA) with 6 MV beam data acquired at 100‐cm SSD, we re‐configured a TBI‐specific dose calculation model using 6 MV beams acquired at the TPS‐permitted maximum 140‐cm SSD and commissioned it at 180‐cm SSD.

The TBI‐specific dose calculation model was configured with measured percentage depth dose (PDD), beam profiles (PRF), diagonal profiles of maximum square field (DPR), and output factors at an SSD of 140 cm. The model was calibrated with the absolute point dose measured at a 180‐cm SSD to align with the TBI treatment scenario as closely as possible, following the recommendations of the AAPM TG‐106 report^22^ and TPS manufacturer guidelines.^23^ The PDDs, PRFs, and DPRs were scanned using two cylindrical ion chambers (CC13, IBA Dosimetry, Schwazenbruck, German) using a 3D water tank (Blue Phantom 2, IBA Dosimetry, Schwazenbruck, Germany). Due to the beam divergence effect, the 40 × 40 cm^2^ field size at 100‐cm SAD would augment to 56 × 56 cm^2^ field size at 140‐cm SSD, which exceeds the scanning ranges of the water tank (478 mm × 478 mm × 410 mm). Therefore, we scanned half of the PRFs and DPRs by shifting the water tank ∼25 cm off the beam central axis and then reconstructed complete PRFs and DPRs symmetrically. When acquiring DPRs, we rotated the collimator to 45° and used the PRFs setting to scan diagonal profiles instead of the DPR setting. The output factors and absolute point dose were measured with 10 × 10 cm^2^ field size and 140‐cm SSD using a calibrated ion chamber (PTW, Freiburg, German), adhering to the AAPM TG‐106 report recommendations.^22^ The MLC transmission factor and dosimetric leaf gap (DLG) were measured using “Ada” and “Chair” fields at 10 cm depth and 180 cm SSD, following the TPS vendor recommendations.^23^

#### 2.2.2 Model commissioning

To verify the accuracy of the TBI‐specific dose calculation model, we conducted an end‐to‐end (E2E) test at 180 cm SSD using a Farmer‐type ion chamber (CC13, IBA Dosimetry, Schwazenbruck, German) with a nominal 30 × 30 cm^2^ field size (actual size at 100 cm SAD, and so far below) in both a solid water phantom and heterogeneous thorax phantom (CIRS anthropomorphic thorax phantom, SunNuclear, Florida, USA). Phantoms were scanned individually using a large‐bore CT simulator (GE Discovery 590 RT, GE Healthcare, Milwaukee, WI, USA) with a 2.5 mm slice thickness. The image dataset was then imported into TPS (Eclipse v.15.1, Varian Medical System, Palo Alto, CA, USA) for subsequent end‐to‐end dose calculation.

We selected six depth points and ten off‐axis points for E2E verification. Point doses at depths of 1.5, 5.0, 10.0, 15.0, 20.0, and 25.0 cm were measured using a calibrated ion chamber (PTW, Freiburg, German) and compared against TPS‐calculated doses at corresponding points. Similarly, ten off‐axis point doses on the collimator X‐axis, at an interval of 10 cm (from central axis up to 100 cm off‐central axis distance, simulating a 200 cm tall patient), were measured at 5 cm deep and compared against TPS dose calculation. Due to the solid water phantom's size exceeding the measurement range, we moved the phantom laterally and rotated the gantry angle 15° and 30°, respectively, for off‐axis dose measurements. However, measurements at 0° gantry angle were performed directly in the CIRS thorax phantom, considering perpendicular delivery for the thorax region during treatment.

Regarding transmission factor and DLG, we used a 2‐D array detector (MatriXX, IBA, Schwazenbruck, Germany) for planar dose collection. We imported two dynamic MLC test fields into TPS: the dynamic “chair” and the AIDA test fields.^24^ The AIDA pattern contains five rectangle fields shaped by MLC, while the “Chair” test^25^ comprises three main parts for evaluating transmission and DLG. All measurements were conducted in an extended SSD setup. A 2D gamma analysis (2%, 2 mm) with a 10% threshold was performed in the global mode.

#### 2.2.3 in‐vivo dose measurement devices and methods

**FIGURE 2:**
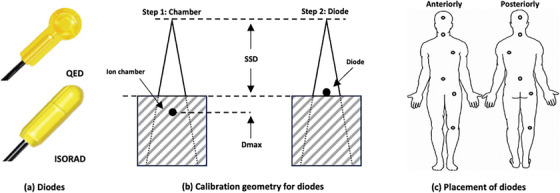
in‐vivo diodes to measure the real‐time surface dose of TBI treatment. (a) diodes (QED for flat volume, ISORAD for cylindrical volume). (b) calibration geometry for in‐vivo dosimetry. (c) Placement of diodes on patients.

For in‐vivo dose monitoring, we employed a wireless SunNuclear IVD 2TM System with two ISORADTM and ten QEDTM diode detectors (SunNuclear, Florida, USA), as illustrated in Figure 2(a). These detectors were calibrated against a 0.6 cm^3^ calibrated ion chamber (PTW, Freiburg, German) at the dose‐maximum depth of 180 cm SSD in a 30 × 30 cm^2^ field size, as shown in Figure 2(b). The QED diode detectors are flat‐designed with active dimensions of 0.8 × 0.8 mm^2^ for easy placement on the patients’ surface, recommended for measuring direct incident beams. ISORAD diode detectors have a cylindrical active volume of 1.4 mm diameter for angular‐independent measurements, suitable for oblique incident beams.

We marked six points (head, neck, lungs, umbilicus, hip, and knee) in the patient's skin during CT simulation, as illustrated in Figure 2 **(c),** and placed diodes (SunNuclear, Florida, USA) on the marked points for real‐time dose monitoring during TBI treatment. Concurrently, a calibrated ion chamber (PTW, Freiburg, German) was placed on the umbilicus for absolute dose reference. Therapists would immediately stop the treatment and contact responsible physicists and oncologists to address significant variations (>5% tolerance) between the real‐time readings and TPS‐calculated dose. Physicists would adapt treatment plans for the remaining fractions based on the in‐vivo readings if necessary.

### 2.3 TBI implementation

#### 2.3.1 Prescription

The prescription dose for TBI is 8–10 Gy, delivered in 4–5 fractions over 2–3 consecutive days, twice daily. The planning target volume (PTV) encompasses the entire body with a 5 mm contraction from the skin and is typically divided into four sub‐parts: head, thorax, abdomen, and legs. The plan criteria require that 90% of the prescription dose covers 95% of the PTV, and the MLD should remain below 7 Gy.

#### 2.3.2 CT simulation

During the CT simulation, patients were immobilized with vacuum bags in both supine and prone positions. Patients lay flatly in each position with their arms along the body. Before scanning, repositioning lines and 12 monitoring points were marked with radiation‐visible markers. The upper and lower body scans were conducted separately and then reconstructed into a whole‐body CT using in‐house software,^26^ due to the CT scanner's range limitation of approximately 160 cm.

#### 2.3.3 TBI planning

All clinical plans were designed at three couch positions using a TBI‐specific dose calculation model in TPS (Eclipse, Varian Medical Systems, Palo Alto, CA). Instead of the original treatment couch, a custom‐designed sliding couch system was employed due to its limited travel range during treatment. The couch angle was set to 90 degrees during planning to mimic the patient's lying position under the gantry, and a TBI‐specific table tolerance was created to accommodate this. Large open fields were used for head and leg irradiations at two different couch positions to improve treatment efficiency. For the thorax and abdomen regions, large fields with MLC shielding were used for lung protection at another couch position. The static MLC shielding was based on the Beam's Eye View (BEV) projection. All field widths were set to 40 cm laterally to account for possible patient intra‐fractional movement. Field junctions of the adjacent fields were shifted to smear hot/cold regions. Upon completion of the planning, oncologists evaluated the plans based on dose‐volume histogram (DVH) for lung‐sparing and dose lines for target coverage.

In the aspect of planning strategies, the main challenges with the open‐field TBI technique are achieving dose homogeneity at field junctions and patient edges due to significant variances in patient thickness. The hot/cold areas in the field junctions could be smeared by shifting the junctions of each field. Optimizing MLC sequencing to shield hot areas at patient extremities might help, though it can affect plan robustness. Adding an appropriate bolus to compensate for changes in the patients’ thickness during treatment can also eliminate hot/cold areas. Additionally, a decent movement tolerance is necessary when setting up field widths to ensure adequate dose delivery. Typically, a ±1 cm setup uncertainty is recommended for planning robustness.

### 2.4 Clinical workflow and quality controls for TBI treatment

The clinical workflow concludes with a multidisciplinary team discussion, baseline assessment of the patient and prescription, patient immobilization and CT scanning, treatment planning in TPS, initial plan check and dosimetry check, treatment preparation, in‐room timeout checklist, beam delivery, in‐vivo monitoring, and follow‐ups after treatment.

To assure the safety of the TBI treatment, we implement several measures such as machine interlocks, pretreatment checklists, and in‐vivo dose monitoring. After planning, a qualified medical physicist reviews all TBI plans according to the TBI‐specific initial plan chart check (Table 1), which includes checks for dose rate, field size, block positions, and TBI‐specific dose calculation model. Physicists also perform a patient‐specific quality assurance (PSQA) by measuring the absolute dose of delivered beams using a calibrated ion chamber (PTW, Freiburg, German) in a solid water phantom before approving the treatment plans.

**TABLE 1:** TBI‐specific initial planning chart checks.

No.	Check items	Status
1	Patient identification and prescription check	
2	Integrity of the planning CT	
3	Planning SSD check	
4	Isocenter placement check (usually at umbilicus)	
5	Patient treatment position checks (FFS, FFP) for each sub‐plan	
6	Check if field's maximum gantry rotation angle ≤ 30 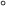	
7	Check if field sizes are enough tolerance for patient movements	
8	Check if using TBI‐specific dose calculation model	
9	Dose rate check (for lungs and abdomen, dose rates ≤100 MU/min)	
10	Check multi‐leaf collimator aperture is shaped for lungs in both AP and PA positions, and sparing MLD ≤7 Gy	
11	Add block to each treatment field	
12	Check if PMMA board needed for planning	
13	If PMMA needed, check density override of the PMMA board	
14	If PMMA needed, state the height of the PMMA board	

Abbreviations: AP, anterior to posterior; CT, computed tomography; FFP, feet‐first prone; FFS, feet‐first supine; MLD, mean lung dose; PA, posterior to anterior; PMMA, polymethyl‐methacrylate; SSD, source‐to‐surface distance; TBI, total body irradiation.

Special attention is given to the safety of the transmission block to prevent risks of falling and misplacement. In our institution, the transmission block is securely screwed onto a TBI‐dedicated block tray which is mounted on the slots, preventing it from falling or inter‐fractional misplacement. During treatment planning, this transmission block is added to each field in TPS with custom coding. Additionally, the maximum gantry rotation angle is limited to 10° for TBI planning to ensure the transmission block's stability. A qualified physicist performs TBI‐specific initial planning chart checks to ensure these measures are correctly implemented. On treatment day, the transmission block is mounted on the slot using TBI‐specific custom coding, and patients are repositioned using external lasers on the wall and ceiling. Two therapists and attending oncologists conduct a TBI‐specific in‐room timeout checklist (Table 2), which includes daily dose checks with the block, 40‐min disinfection by ultraviolet light, setup of the sliding couch and vacuum bags, patient repositioning with wall and ceiling lasers, placement of in vivo monitoring devices, and SSD checks. On the treatment console, therapists must verify the block placement again before the beam is turned on; otherwise, the beam will be automatically interlocked. Once everything is confirmed, therapists start the treatments and monitor the real‐time readings. If more than a 5% dose variation is detected, therapists immediately stop the beam and contact attending physicists and oncologists to address the issues.

**TABLE 2:** TBI‐specific in‐room timeout checklist.

No.	Roles	Check items	Status
1	Nurses	Room disinfection by ultraviolet light for 40 min	
2	Therapists	Planning checks and LINAC preparation	
3	Therapists/Physicists	Position TBI custom‐designed sliding couch with external lasers	
4	Therapists/Physicists	Check the usage and height of the PMMA board	
5	Therapists/Physicists	Check placement of transmission block and machine interlocks	
6	Therapists/Physicists	Reposition the patient	
7	Therapists/Physicists	SSD checks	
8	Physicists	Placement and connection of in‐vivo dose measurement devices	
9	Therapists	Checked transmission block interlocks on the console	
10	Therapists	Beam on	

Abbreviations: LINAC, linear accelerator; PMMA, polymethyl‐methacrylate; SSD, source‐to‐surface distance; TBI, total body irradiation.

After TBI treatments, the toxicity is evaluated during follow‐ups according to toxicity criteria from the Radiation Therapy Oncology Group and the European Organization for Research and Treatment of Cancer (RTOG/EORTC).^28^

## 3 RESULTS

### 3.1 Instantaneous dose rates after the transmission block

As SSD varies from 185 to 200 cm, the instantaneous dose rates were around 7 cGy/min at patients’ mid‐plane (15.0/12.5/10.0/7.5 cm), as tabulated in Table **3**. The beam quality index (TPR_20_/TPR_10_) of the block‐filtered 6 MV x‐rays is 0.695, compared to 0.670 for the un‐blocked 6 MV beam at 100 cm SAD with 10 × 10 cm^2^ field size. The quantifies the beam hardening effect induced by the transmission block. As the beam hardens, the build‐up region might expand, necessitating consideration of the absorbed dose in the shallow area, which implies potential usage of the PMMA board.

**TABLE 3:** The instantaneous dose rates after transmission block with variable source‐to‐chamber distances (cm).

Source‐to‐chamber distance (cm)	185.0	190.0	195.0	200.0
Depth (cm)	15.00	12.50	10.00	7.50
Measured point dose at patients’ midplane (cGy)	7.08	6.75	7.02	7.30

### 3.2 Commissioning of TBI‐specific dose calculation model

#### 3.2.1 TPS modeling

The absolute dose of block‐filtered 6 MV x‐rays at 5 cm depth with a 10 × 10 cm^2^ field size is 763.744 MU for 1 Gy at 180 cm SSD (as compared to 100 MU for 1 Gy without transmission lead at 100 cm SSD). Compared to the previous model, the new output factors for various field sizes increased, indicating enhanced scattering caused by the transmission block.

**FIGURE 3:**
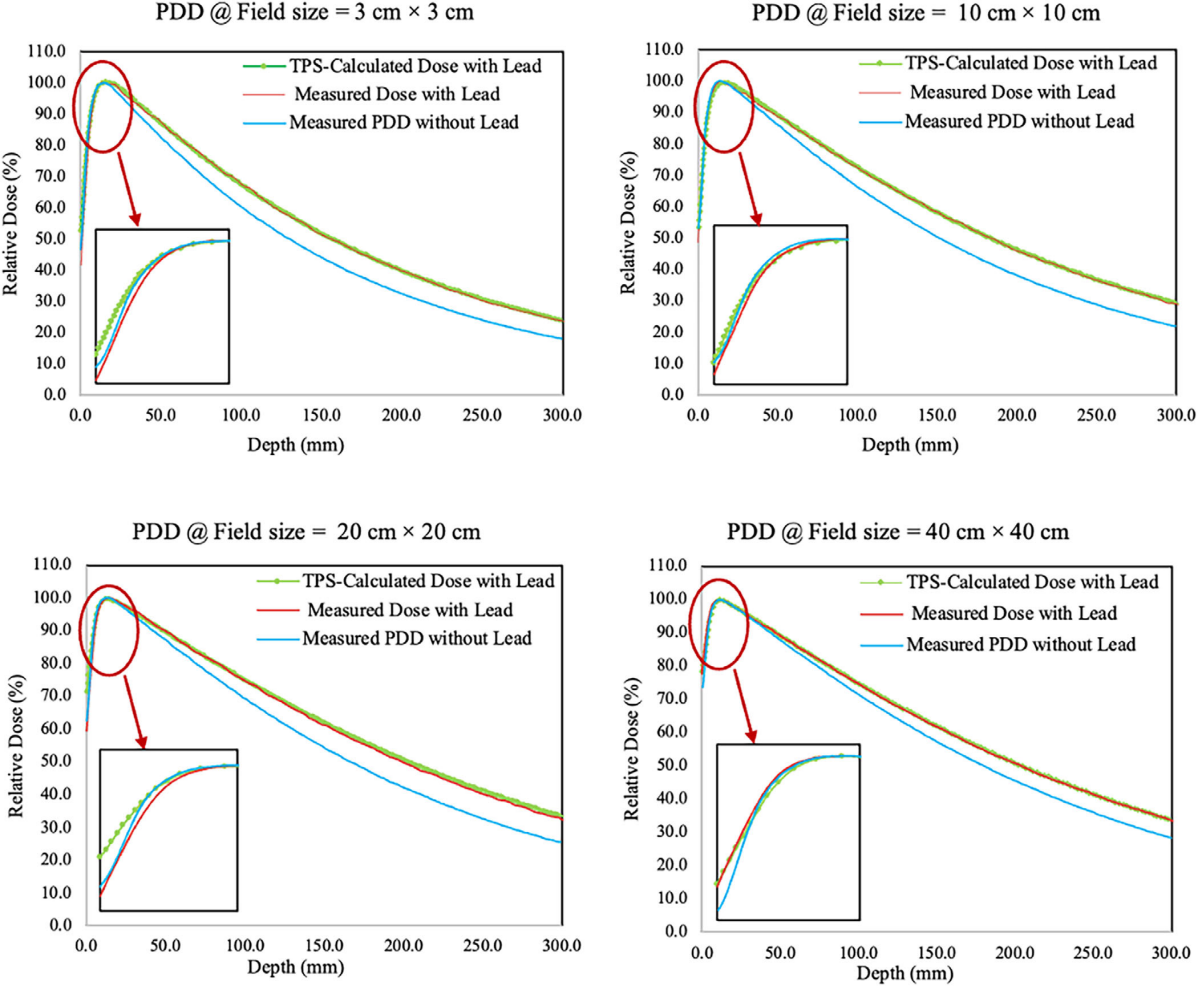
Percentage depth doses (PPDs) at various field sizes. Green lines represented PDDs of 6 MV x‐rays calculated by a reconfigured TBI‐specific dose calculation model with transmission lead; red lines represented PDDs of 6 MV x‐rays measured by chambers behind the transmission lead; and blue lines represented PDDs measured by chambers without transmission lead.

**FIGURE 4:**
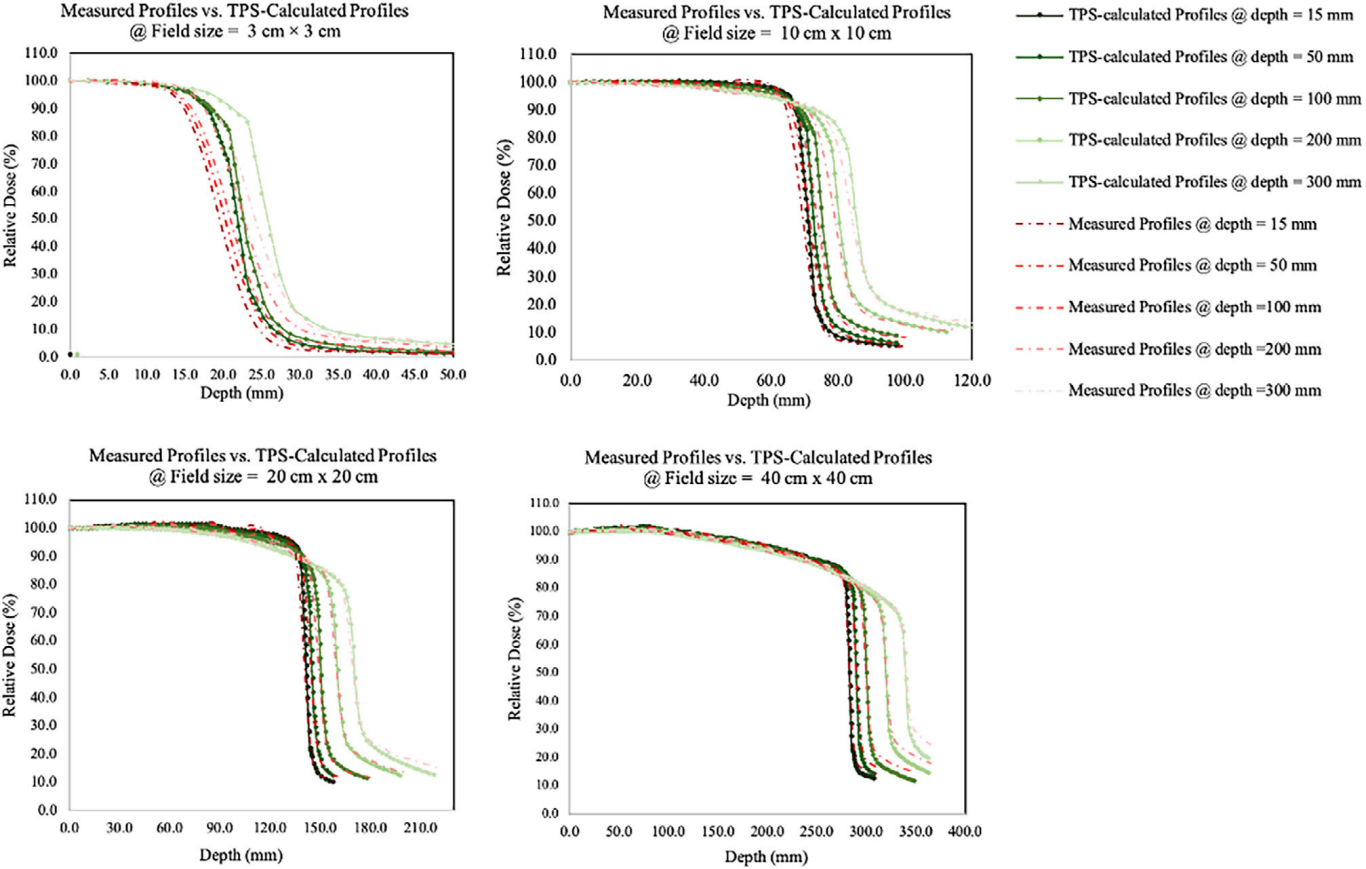
Beam profiles (PRFs) at various field sizes. Green lines represented PRFs of 6 MV x‐rays that were calculated by a reconfigured TBI‐specific dose calculation model with transmission lead at depths of 15, 50, 100, 200, and 300 mm; red lines represented PRFs of 6 MV x‐rays that were measured by chambers behind the transmission lead at depths of 15, 50, 100, 200, and 300 mm.

**Figures** **3** and **4** present the measured PDDs and PRFs. The PDDs for 6 MV x‐rays with transmission lead consistently increased after the depth of maximum dose (d_max_) compared to 6 MV x‐rays without transmission lead. When comparing measured PDDs with transmission lead against TPS‐calculated PDDs with the transmission lead, the variance was primarily observed in the build‐up regions before d_max_. For PRFs, there was a noticeable difference between measurements with transmission lead and TPS‐calculated doses with transmission lead with for a 3 cm × 3 cm field size. However, there were few differences in data with other field sizes.

Our TBI setting demonstrated a transmission factor of 0.0114 and a DLG value of 3.17 mm (compared to 0.0135 and 1.80 mm for standard treatment distances). Compared to the standard TPS model at 100 cm SSD for 6 MV X‐rays, the transmission factor decreased by approximately 15.6%, and DLG increased by 76.1%. These changes in DLG, induced by the transmission block, are challenging to quantify due to the involvement of multiple factors such as the beam hardening effect and the extended measurement distance.

#### 3.2.2 E2E verification

AAPM MPPG^27^ recommends a 2% tolerance for the average difference between measured and TPS‐calculated doses in the low‐gradient target region for typical SSDs. In this study, the discrepancy between the TPS‐calculated dose and measurements using a calibrated ion chamber (PTW, Freiburg, German) ranges from 0.22% to 1.47% for depth dose and off‐axis dose in a solid water phantom, as shown in **Tables** **4** and **5**. Although the solid water phantom validation was conducted at the extended SSDs, our results still fall within AAPM MPPG recommended tolerance.

**TABLE 4:** Depth dose validation results on solid water phantom.

Depth (cm)	1.5	5.0	10.0	15.0	20.0	25.0
D_phantom_ (cGy)	10.47	9.62	8.145	6.785	5.59	4.54
D_TPS_ (cGy)	10.4	9.5	8.1	6.8	5.6	4.6
Dose difference (%)	0.67%	1.25%	0.55%	−0.22%	−0.18%	−1.32%

*Note*: D_Measured_, dose measured by ion chamber in the solid water phantom; D_TPS_, TPS‐calculated dose using TBI‐specific model.

**TABLE 5:** Off‐axis dose validation results on solid water phantom.

Gantry angle (°)	0			15			30				
Off‐axis distance (cm)	0	10	20	30	40	50	60	70	80	90	100
D_phantom_ (cGy)	6.72	6.78	6.67	6.35	6.26	5.77	5.34	5.07	4.91	4.41	3.35
D_TPS_ (cGy)	6.70	6.70	6.58	6.40	6.20	5.7	5.40	5.15	4.85	4.40	3.40
Dose difference (%)	0.30	1.19	1.37	0.78	0.97	1.23	1.11	1.55	1.24	0.23	1.47

*Note*: D_Measured_, dose measured by ion chamber in the solid water phantom; D_TPS_, TPS‐calculated dose using TBI‐specific model.

For E2E verification using a CIRS phantom, AAPM MPPG recommends a 5% relative dose difference for IMRT commissioning with the photon beams.^27^ In this study, our results (Table **6**) demonstrated a 1.2%–4.85% difference in dose to lungs, spinal cord, and normal tissues, indicating that the TBI‐specific dose calculation model demonstrated acceptable accuracy when used for calculating TBI plans with a transmission block at extended SSDs of 180–200 cm.

**TABLE 6:** End‐to‐end (E2E) testing in anthropomorphic CIRS Thorax phantom.

Calculated and measured condition	Gantry angle = 0°, MU = 200, SSD = 180 cm, Field size = 30 × 30 cm^2^					
Number of points	1	2	3	4	5	6
Materials	Tissue	Tissue	Tissue	Tissue	Spinal cord	Lungs
Off‐axis distance to the central axis (cm)	0	0	0	4	0	8
Depth of points (cm)	7.0	3.0	10.0	7.0	15.0	10.0
D_Measured_ (cGy)	8.83	10.51	7.64	9.06	5.83	9.51
D_TPS_ (cGy)	8.60	10.00	7.90	8.70	5.90	9.10
Dose difference (%)	2.60	4.85	3.40	3.97	1.20	4.31

*Note*: D_Measured_, dose measured by ion chamber in CIRS thorax phantom; D_TPS_, TPS‐calculated dose using TBI‐specific model.

Abbreviations: MU, monitor unit; SSD, source‐to‐surface distance.

### 3.3 Plan parameters

**FIGURE 5:**
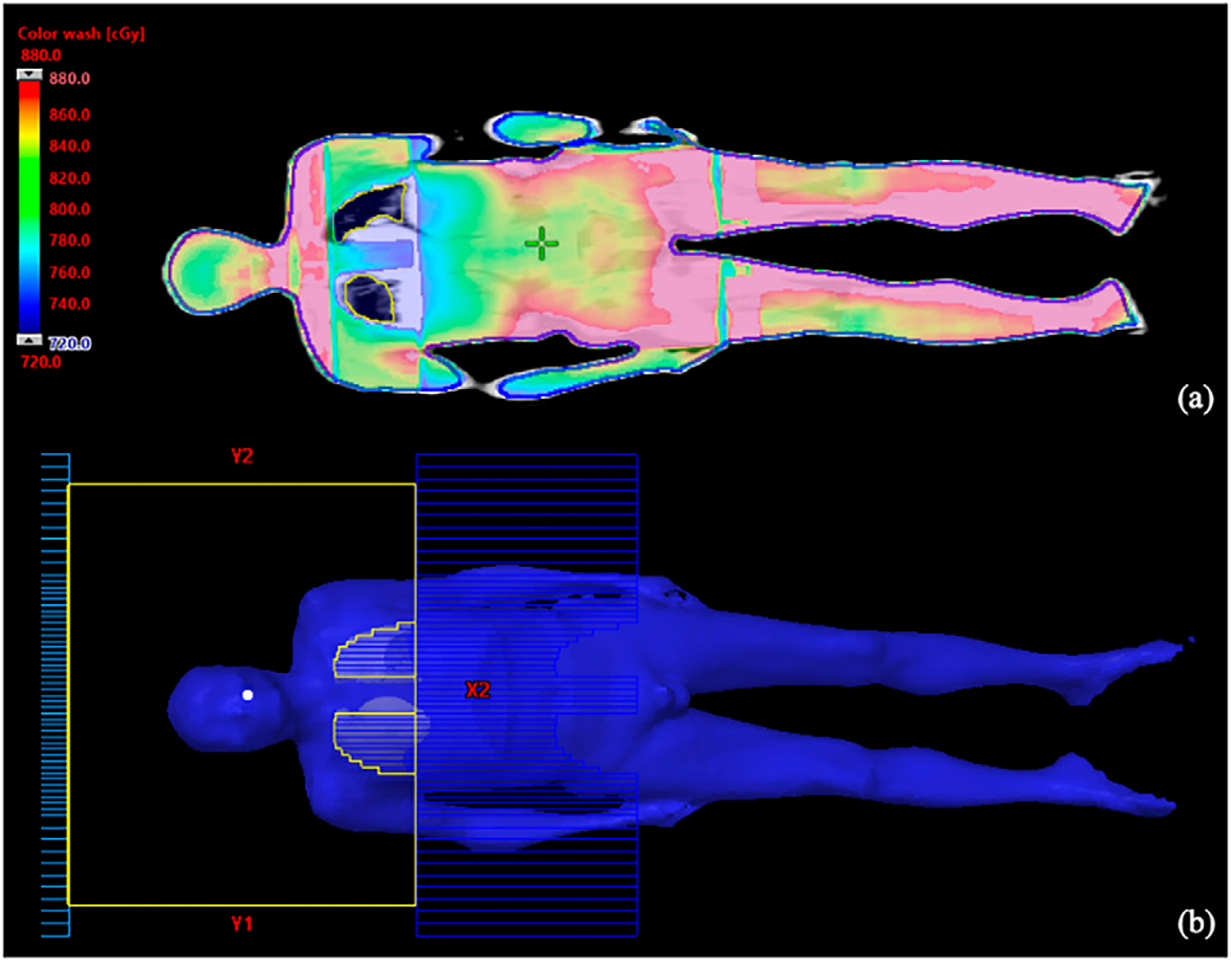
Dose distribution of TBI plans. (a) dose distribution for multi‐center open‐field TBI technique; (b) Open field with MLC shielding for lungs.

The dose distribution of the TBI planning is illustrated in Figure **5**. The PTV coverage outside the lung regions is V_90%PD_ (PTV) ≈ 95%, V_115%PD_ (PTV) ≤ 4.0%. For lung protection, the MLD ≈ 6.8 Gy, D_95%_ ≈ 6.0 Gy, and D_max_ ≈ 8.0 Gy (Table **7**).

**TABLE 7:** Dose distribution of open‐field TBI technique.

Techniques	Open‐field TBI technique
Planning time	∼ 60 min
Calculation algorithm	TBI‐specific dose calculation model
PTV without lungs	V_90%PD _≈ 95%, V_115%PD_ ≤ 5.0%
Lungs	MLD ≈ 6.8 Gy, D_95%_(lungs) ≈ 6.0 Gy, D_max_(lungs) ≈ 8.0 Gy
Hot areas	Field junctions and body edges
Cold areas	Field junctions and tips of the toes
Delivery time	∼ 50 mins

*Note*: V_90%PD_, target volume receiving 90% prescription dose; V_115%PD_, target volume receiving 115% prescription dose; D_95%_(lungs), the dose received by 95% lung volume; D_max_(lungs), the maximum dose in lungs.

Abbreviations: MLD, mean lung dose; PTV, planning target volume; TBI, total body irradiation.

Cold areas were identified in two regions: one in the upper thoracic regions as a trade‐off for lung protection, which still ensured about 5.8–6.0 Gy dose coverage, and the other at the tips of the toes, which could be improved using a 5 mm bolus. In contrast, hot areas are mainly concentrated around the edges of the limbs due to the apparent thickness decrease compared to the surrounding tissues but still maintained V_115%_ ≤ 5%. Besides, the final dose distribution indicated that an additional PMMA board is not required.

Regarding planning and delivery efficiency, a TBI plan with the TBI‐specific dose calculation model took about 1 h to complete, and beam delivery took about 50 min.

### 3.4 In‐vivo dose monitoring and follow‐ups

Thirty patients received hyper‐fractionated TBI (8–10 Gy in 4–5 fraction, BID, over three consecutive days) utilizing the open‐field TBI technique. All fractions were monitored in real‐time using six diodes and one calibrated ion chamber (PTW, Freiburg, German) in prone and supine positions, respectively. As compared to the TPS‐calculated dose in treatment planning, the real‐time readings demonstrated within 5% mean relative difference from (MRD) from head to patella, as stated in Table 8, meeting AAPM requirements for clinical treatment.^27^ After treatment, we followed these patients regularly to evaluate their lung toxicity for 6–12 months and found that no patients experienced grade 2 or higher radiation pneumonitis, based on RTOG/EORTC criteria.^28^

**TABLE 8:** in‐vivo dose monitoring results using diodes and calibrated ion chamber.

PA positions	Head	Neck	Left lung	Umbilicus	Hip	Patella	Ion chamber
Dose MRD (%)	−1.49 ± 2.41	0.10 ± 1.83	−0.66 ± 2.31	−0.45 ± 3.27	−1.39 ± 2.71	2.10 ± 2.15	−1.55 ± 2.11

AP Position	Head	Neck	Left Lung	Umbilicus	Hip	Patella	Ion chamber
Dose MRD (%)	−1.49 ± 1.84	0.21 ± 2.85	−2.59 ± 2.76	−1.59 ± 2.64	−1.38 ± 3.03	0.40 ± 3.55	−3.42 ± 1.51

Abbreviation: AP, anterior‐to‐posterior; MRD, mean relative difference, which is expressed as mean relative error ± mean relative standard deviations; PA, posterior‐to‐anterior.

## 4 DISCUSSION

This is the first study to configure a TBI‐specific dose calculation model using beam data acquired at SSD of 140 cm and apply it to TBI treatment at SSDs ranging from 180 to 200 cm. In contrast, some studies^29^ applied the standard dose calculation model (configured at 100 cm SSD) to TBI treatment at extended SSDs after some commissioning procedures. However, that does not work in our cases. However, the goal of reducing PT after TBI treatment promoted us to employ a lead transmission block to reduce dose rates down to ≤10 cGy/min at the patients’ midplane at 180−200 cm SSDs, leading to alterations in beam properties. Consequently, we reconfigured a TBI‐specific dose calculation model for 6‐MV x‐rays at a TPS‐permitted maximum SSD of 140 cm, balancing the TBI treatment scenarios with the requirements of the TPS. LINCAs with lower meter‐set dose rates may not necessitate an additional transmission block, thus obviating the need for subsequent commissioning procedures.

Configuration of a TBI‐specific model at an extended distance is challenging since the field size there has been beyond the measurement range of the water tank. We have to segment the beam scanning and reconstruct them to complete PRFs and DPRs. Also, the methodologies to determine the DLG values at a 180 cm SSD could be further investigated even though the current DLG setting demonstrated acceptable performance with our TBI technique based on current clinical results, possibly because we did not employ inverse‐modulated optimization. Nevertheless, the dose difference is within 5% in the E2E verification, meeting the AAPM recommendations.^27^ Also, some studies directly employed a 600 MU/min instantaneous dose rate to irradiate patients at around 100 cm SSD^30^, ^31^, ^32^, ^33^, ^34^ but there are no follow‐up results for us to refer to. We still cannot take the risk to level up the instantaneous dose rates at the beginning but it could be taken into consideration in the future. So far, we have monitored 30 patients for PT over 6 months. No patients experienced grade 2 or higher radiation pneumonitis, accounting for RTOG/EORTC toxicity criteria,^28^ which results from the combination of both a low instantaneous dose rate (≤10 cGy/min) and a low total dose to the lungs (MLD ≤ 7 Gy).

There are some limitations in our study. First, the prescription of the hyper‐fractionated TBI (8–10 Gy) in our center is relatively lower as compared to others (typically 10–12 Gy).^9^ It might be another reason contributing to the low incidence of PT due to fractionation changes. Also, lung shielding usually causes unexpected dose heterogeneity in the thoracic regions, which might increase the risk of transplantation failure. A sequent boost dose^35^, ^36^ should be considered in case of marrow residue. For instance, Hansen^35^ incorporated VMAT or IMRT boosts in four out of seven fractions to thoracic regions at standard SSDs combined with extended‐SSD TBI treatment as a strategy to address this issue.

## 5 CONCLUSIONS

A low instantaneous dose rate TBI technique can be implemented in the clinic.

## AUTHOR CONTRIBUTIONS

Zhengxin Gao contributed to all aspects of the research and manuscript preparation. Qiuyi Xu and Qing Gong were responsible for the configuration and validation of TBI‐specific TPS models, and the implementation of TBI. Dr. Fengjiao Zhang and Dr. Qiaoying Hu focused on the clinical aspects of TBI treatment. Yaling Hong conducted in‐vivo dose monitoring. Dr. Qi Yu carried out the follow‐up analysis of post‐TBI patients. Qing Gong and Dr. Shen Fu supervised the research, with Qing Gong overseeing the physics aspects and Dr. Shen Fu overseeing the medical aspects.

## CONFLICT OF INTEREST STATEMENT

This study was supported by grants from the research project of Shanghai Huangpu District Health System (No. HLZ202218); the Project of State Key Laboratory of Radiation Medicine and Protection, Soochow University (No. GZK1202131); the Guangzhou High‐tech, Key, and Characteristic Clinical Technology of the Guangzhou Municipal Health Commission (grant number: 2023C‐TS64); the National Natural Science Foundation of China (81773225).
